# Complete Genome Sequencing of Campylobacter jejuni Strain X Reveals the Presence of pVir- and pTet-like Plasmids

**DOI:** 10.1128/mra.00421-22

**Published:** 2022-06-28

**Authors:** Andrey V. Karlyshev

**Affiliations:** a School of Life Sciences, Pharmacy, and Chemistry, Faculty of Science, Engineering, and Computing, Kingston University London, Kingston upon Thames, United Kingdom; Queens College CUNY

## Abstract

Here, I report the complete genome sequence of Campylobacter jejuni strain X, containing two plasmids similar to pVir and pTet, which were originally identified in strain 81-176. Scrutiny of complete genome sequences in GenBank revealed several other strains with similar plasmid contents. Comparative genome analysis suggested a common origin of these plasmids.

## ANNOUNCEMENT

Campylobacter jejuni strain X (NCTC13357), which was isolated from a person with bloody diarrhea in London, United Kingdom, in 2001, is untypeable (1) but capable of producing a capsule (2, 3), the genetic origin of which was investigated (4, 5). A draft sequence of its genome published in 2014 (GenBank accession number GCA_000466925.2) did not allow the identification of plasmids, which became possible via the compete genome sequencing reported here.

The strain was grown on brain heart infusion agar (Oxoid, USA) for 48 h at 37°C in a microaerobic atmosphere. Approximately 10^8^ CFU were suspended in 120 μL of Tris-EDTA (TE) buffer with lysozyme (0.1 mg/mL) and RNase A (0.1 mg/mL) and incubated for 25 min at 37°C, followed by the addition of proteinase K and SDS to 0.1 mg/mL and 0.5%, respectively, and incubation for 5 min at 65°C. Genomic DNA was purified using an equal volume of SPRI beads (Beckman, USA) and resuspended in elution buffer (EB) (Qiagen, Germany).

Preparation of the DNA sequencing library was performed using the Nextera XT library preparation kit (Illumina, San Diego, CA, USA). Paired short reads (2 × 279,287 reads) (average sizes of 235 bp and 245 bp per read) produced by the Illumina NovaSeq 6000 system (NovaSeq 6000 SP sequencing kit v1.5 [2 × 250 bp]) were assembled using SPAdes v3.7 into 16 contigs (1 to 380,853 kb, with an *N*_50_ value of 226,489 bp) with 74.88× coverage. Long-read genomic DNA (unsheared) libraries were produced using the Oxford Nanopore Technologies (ONT) SQK-LSK109 kit with the native barcoding EXP-NBD104/114 kit. The barcoded sequencing library was run in a FLO-MIN106 (R.9.4.1) flow cell on a GridION system (ONT). After adapter trimming with Trimmomatic v0.30 with a sliding window quality cutoff score of Q15 (6), 14,764 long reads (up to 164 kb, with an *N*_50_ value of 14,211 bp) combined with short Illumina reads were used in a hybrid assembly by Unicycler v0.4.9b with default parameters (7). The assembly was polished using Illumina reads. The assembly coverage with Illumina and Nanopore reads was 74.34× and 116.22×, respectively.

The total genome assembly size was 1,805,220 bp, including a chromosome (1,723,526 bp) and two plasmids, i.e., pVir (35,898 bp) and pTet (45,796 bp), with a GC content of 30.33%. Genome sequence annotation was performed by NCBI Prokaryotic Genome Annotation Pipeline (PGAP) v5.1 (8), using the best-placed reference protein set and GeneMarkS-2+, which identified 1895 genes, including 1,775 protein-coding genes and 64 pseudogenes, in addition to 56 genes, encoding 44 tRNAs, 3 sets of rRNA (5S, 16S, and 23S), and 3 noncoding RNAs.

**FIG 1:**
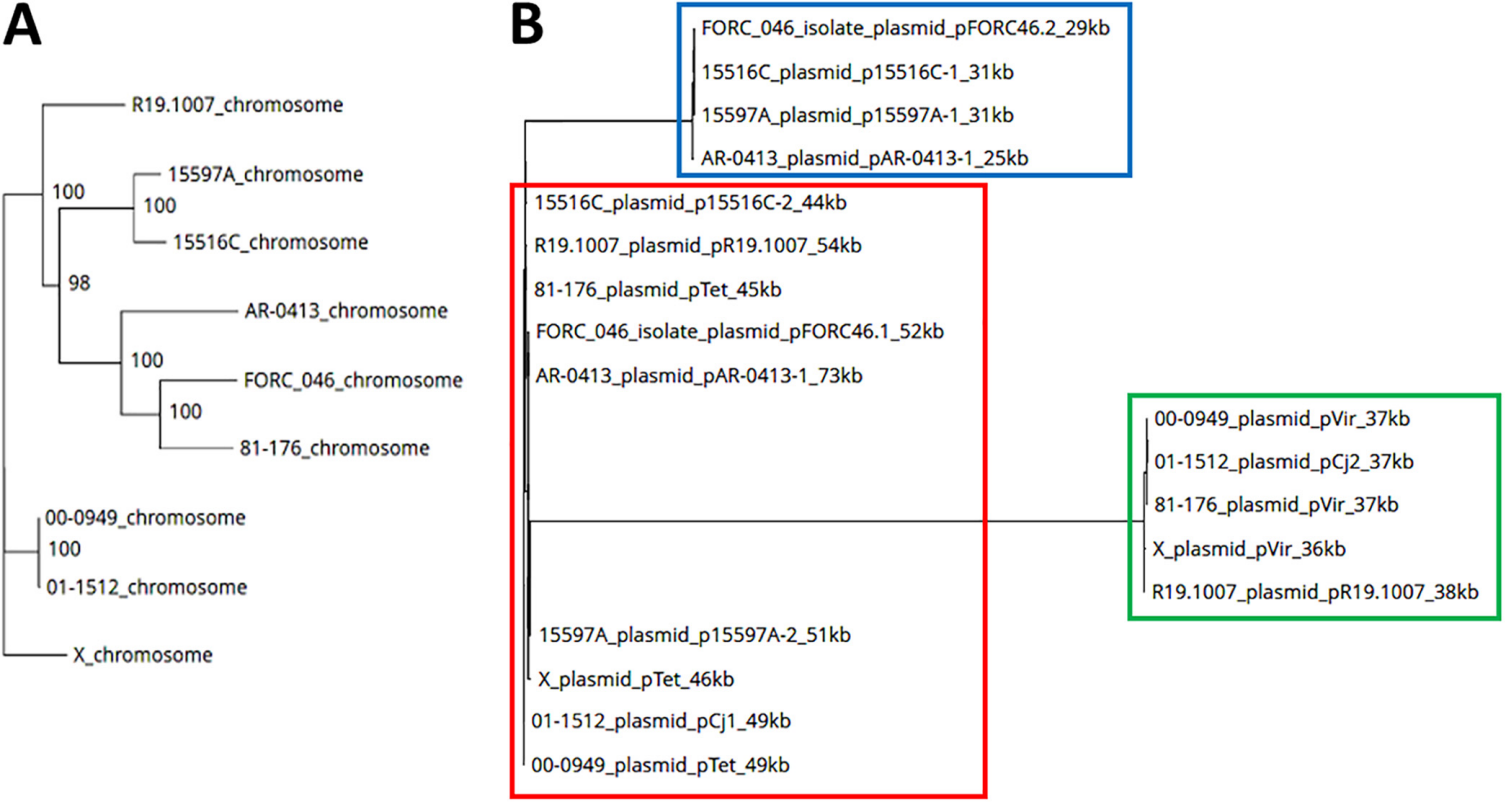
Core genome analysis of chromosomes (A) and plasmids (B) of selected strains of C. jejuni, i.e., R19.1007 (GenBank accession number GCA_019056455.1), AR-0413 (GenBank accession number GCA_008727455.1), FORC_046 (GenBank accession number GCA_002214785.1), 15516C (GenBank accession number GCA_022220425.1), 00-949 (GenBank accession number GCA_000835365.1), 01-1512 (GenBank accession number GCA_000835345.1), 15597A (GenBank accession number GCA_022220325.1), 81-176 (GenBank accession number GCA_000015525.1), and X (GenBank accession number GCA_000466925.3). Red, green, and blue boxes in panel B represent clades of sequences related to pTet, pVir, and cryptic plasmids, respectively. Settings were as follows: maximum E value cutoff, 0.01; identity minimum percentage cutoff, 40%; minimum percentage for core, 50%; 100 bootstrap iterations applied. An overview of the M1CR0B1AL1Z3R algorithm (11) is available at https://microbializer.tau.ac.il/overview.html.

A detailed comparative sequence analysis of plasmids found in Campylobacter spp. was performed previously (9). For the purpose of this study, we selected only strains containing two plasmids with sizes similar to those of pVir and pTet plasmids originally detected in strain 81-176 (10). Scrutiny of complete genome sequences in GenBank revealed nine strains containing such plasmids. Core genome analysis using the M1CR0B1AL1Z3R tool (11), which was conducted separately for chromosomes (Fig. 1A) and plasmids (Fig. 1B), revealed clustering of the latter into several clades, suggesting their common origin. Remarkably, while all strains contain pTet-like plasmids, pVir-like plasmids are present in only five of them, with the remining clades containing cryptic plasmids of relatively small sizes. Much greater variation in the sizes of pTet-like plasmids, compared with pVir-like plasmids (44 to 73 kb and 36 to 38 kb, respectively), may also suggest an earlier origin of the former. This hypothesis requires further investigation.

### Data availability.

This whole-genome project has been deposited in DDBJ/EMBL/GenBank under the assembly accession number GCA_000466925.3 (chromosome, GenBank accession number CP076835.1; pTet, GenBank accession number CP076836.1; pVir, GenBank accession number CP076837.1), BioProject accession number PRJNA213994, and BioSample accession number SAMN02298816. Raw sequences have been deposited under SRA accession numbers SRR18942393 (long reads) and SRR18942394 (short reads).
